# Impact of weathered multi-walled carbon nanotubes on the epithelial cells of the intestinal tract in the freshwater grazers *Lymnaea stagnalis* and *Rhithrogena semicolorata*

**DOI:** 10.1007/s11356-022-22225-3

**Published:** 2022-07-28

**Authors:** Katrin Weise, Thomas Kurth, Anna Schmidt, Carola Winkelmann, Jochen Becker, Susanne Kretschmar, Thomas Ulrich Berendonk, Dirk Jungmann

**Affiliations:** 1 Faculty of Environmental Sciences, Institute for Hydrobiology, Technische Universität Dresden, Zellescher Weg 40, 01217 Dresden, Germany; 2 Center for Molecular and Cellular Bioengineering (CMCB), Technology Platform, Technische Universität Dresden, Fetscherstraße 105, 01307 Dresden, Germany; 3 Institute for Integrated Natural Sciences, University of Koblenz-Landau, Universitätsstraße 1, 56070 Koblenz, Germany

**Keywords:** Histology, wMWCNTs, Digestive gland, Midgut lumen, Epithelial cells, Physiological state

## Abstract

Freshwater grazers are suitable organisms to investigate the fate of environmental pollutants, such as weathered multi-walled carbon nanotubes (wMWCNTs). One key process is the uptake of ingested materials into digestive or absorptive cells. To address this, we investigated the localization of wMWCNTs in the intestinal tracts of the mud snail *Lymnaea stagnalis* (*L. stagnalis*) and the mayfly *Rhithrogena semicolorata* (*R. semicolorata*). In *L. stagnalis*, bundles of wMWCNTs could be detected in the midgut lumen, whereas only single wMWCNTs could be detected in the lumina of the digestive gland. Intracellular uptake of wMWCNTs was detected by transmission electron microscopy (TEM) but was restricted to the cells of the digestive gland. In larvae of *R. semicolorata*, irritations of the microvilli and damages in the apical parts of the epithelial gut cells were detected after feeding with 1 to 10 mg/L wMWCNTs. In both models, we detected fibrillar structures in close association with the epithelial cells that formed peritrophic membranes (PMs). The PM may cause a reduced transmission of wMWCNT bundles into the epithelium by forming a filter barrier and potentially protecting the cells from the wMWCNTs. As a result, the uptake of wMWCNTs into cells is rare in mud snails and may not occur at all in mayfly larvae. In addition, we monitor physiological markers such as levels of glycogen or triglycerides and the RNA/DNA ratio. This ratio was significantly affected in *L. stagnalis* after 24 days with 10 mg/L wMWCNTs, but not in *R. semicolorata* after 28 days and 10 mg/L wMWCNTs. However, significant effects on the energy status of *R. semicolorata* were analysed after 28 days of exposure to 1 mg/L wMWCNTs. Furthermore, we observed a significant reduction of phagosomes per enterocyte cell in mayfly larvae at a concentration of 10 mg/L wMWCNTs (*p* < 0.01).

**Supplementary Information:**

The online version contains supplementary material available at 10.1007/s11356-022-22225-3.

## Introduction

Macroinvertebrates such as *L. stagnalis* and *R. semicolorata* are important species that feed on benthic biofilms (Winkelmann et al. 2007; Rybicki et al. 2012). These aquatic biofilms are known to be sinks for many contaminants (Ferguson et al. 2008; Velzeboer et al. 2011), including weathered multi-walled carbon nanotubes (wMWCNTs).

After the uptake of wMWCNTs into the digestive tract of grazing organisms, the fate of these compounds is still largely unknown, which is, however, a very important aspect, as the gut has been described as a barrier for CNTs and other nanomaterials (Van der Zande et al. 2020). It is still not clear whether wMWCNTs can reach the epithelial cells of the intestine and digestive glands and further into other tissues. To our knowledge, only a few studies described the effects of MWCNTs on intestinal tissues. Acute aquatic toxicity tests were performed with *Daphnia magna* (*D. magna*) and revealed agglomerated MWCNTs in the intestinal lumen (Edington et al. 2010). In case of the sediment dwelling oligochaete *Lumbriculus variegatus* (*L. variegatus*), nanotubes occurred in the gut together with the sediment, but they were not absorbed into the gut lining cells (Petersen et al. 2008). In the study of Edington et al. (2010), the wMWCNTs passed the membrane barrier of the epithelial cells but did not get further into the tissue (or other organs). In our study, we investigated the barrier function of the midgut and digestive gland of *L. stagnalis* and the midgut of *R. semicolorata* larvae, thus providing more information about the fate of wMWCNTs.

The mud snail *L. stagnalis* is a frequently and important benthic grazer (Elliot et al. 1988). In a previous study, we demonstrated wMWCNTs in the lumen of the intestinal tract of *L. stagnalis* but did not find them within the epithelial cells of the midgut (Weise et al. 2021). In snails, the main place of nutrient digestion and resorption, however, is the digestive gland, which makes it a more promising site of wMWCNT uptake. It is composed of branched digestive tubules covered with epithelial cells. The cells carry numerous microvilli forming a brush border. The epithelium of the digestive gland consists of three main cell types, namely, digestive, excretory and thin cells (Desouky 2006; Elangovan et al. 2000; Arni 1974). The vacuoles of digestive cells, described as B-cells, are filled with different types of phagocytic granules, e.g., small (SG) or the large green (GG) granules (Arni 1974).

The mayfly *R. semicolorata* (Ephemeroptera; Heptageniidae) belongs to the scraper-feeding group (Cummins 1973) and inhabits riffle sections of rivers and streams where it clings to the benthic substrate and swims in short bursts. The alimentary canal of Ephemeroptera has been described in detail by Boudou et al. (1991), Harker (1997) and Gaino et al. (1997), but similar to other insects, the digestive tract of *R. semicolorata* consists of fore-, mid- and hindgut. The fore- and hindgut are lined with cuticle, whereas the midgut is free of cuticle. There are two borders marking the midgut: the cardiac valve, which is an enlargement marking the transition passage between fore- and midgut, and the Malpighian tubules that mark the transition passage between midgut and posterior hindgut. The epithelium of the midgut consists of two distinct cell types: the digestive cells, which are columnar and ordered side by side along the lumen. The others are regenerative and endocrine cells which are isolated and less frequent (Santos et al. 2018).

Another important aspect for grazers is the protection of the intestinal mucosa from hazardous substances and possible mechanical impacts, especially of the midgut epithelium. In insects and other arthropods, the food bolus in the midgut is surrounded by a non-cellular envelope, the peritrophic membrane (PM), which often contains chitin fibrils and proteins and represents the first barrier against pathogens or hazardous particles (Peters 1967, 1969, 1992; Zhu et al. 2011; Salvador et al. 2014). Furthermore, recent studies showed that the midgut epithelium produces a variety of detoxification enzymes (Zhu et al. 2011; Salvador et al. 2014). PM lies between the food particles in the lumen and the epithelium of the midgut. In the mayfly *Hexagenia* spp., it forms an extracellular sheath membrane (Waller et al. 2016), which is formed by the digestive cells of the midgut (Gaino et al. 1997; Harker 1997; Boudou et al. 1991). This membranous or filamentous structure consists of an organized lattice of proteins, chitin and glycoproteins in a proteoglycan matrix (Peters 1992; Hegedus et al. 2009; Lehane 1997). Others described it as a semipermeable and envelope-like structure lining the midgut and containing proteins and chitin (Wigglesworth 1972; Brandt et al. 1978; Adang and Spence 1981; Ryerse et al. 1992). Therefore, molecules can generally pass through this membrane. Still, it serves as a protective barrier and has been described as a line of defines against toxins and pathogens. In some reviews, the PM is classified into different structures and types. Type I is formed from the midgut epithelium, and type II defines PM as secreted from the cardia (Terra 1990; Terra 2001; Lehane 1997; Hegedus et al. 2009). Type I PM is constantly present in insects such as Dictyoptera, Coleoptera, Hymenoptera, Odonata, Orthoptera, Ephemeroptera and Phasmida. In molluscs, reports on PM are rare; a PM has been shown in the gastropod *Megathura crenulata* (Valk et al. 2014). Therefore, PM filter functions may be crucial to understand the transport mechanisms of wMWCNTs in the lumen of *L. stagnalis* and *R. semicolorata.*

Furthermore, enzymatic processes are also very useful tools to investigate the behaviour of nanomaterials in organisms (Girardello et al. 2015). Todt and Salvini-Plawen (2003) explained that digestive cells contain organelles, and they are involved in intracellular digestion, including endosomes and phagosomes. These store lipids and glycogen, among other substances. Phagosomes are contained in phagocytes, which kill and enzymatically degrade particles such as bacteria or other components. Phagocytosis in enterocytes is part of the resorption process, and changes in the amount of phagocytic granules might serve as an indicator of the efficiency of nutrient uptake and provide hints for cellular stress. We count the number of phagosomes in the epithelial cells of the midgut of *R. semicolorata* to investigate sub-lethal effects of wMWCNTs. In addition, physiological markers such as glycogen, TG and the RNA/DNA ratio of both organisms have been studied to determine any correlations between their physiology and histology.

Little is known about the actual inputs of MWCNTs into environmental compartments despite increasing use of nanomaterials in numerous areas such as drug delivery, textiles, health products, or the automotive industry (Baun et al. 2008; Schwirn and Völker 2016; Bianco et al. 2005). Moreover, MWCNTs in particular become more and more important in the nanomaterial industry (Sebastian et al. 2014), which may lead to further applications in various fields. Occurrence in sediments up to 1 mg/kg is described (Selck et al. 2016). Higher concentrations found in sediments compared to wastewater (3.69–32.66 ng/L, Maurer-Jones et al. 2013) can be explained by agglomeration and sedimentation (Chen et al. 2010; Schierz et al. 2014; Glomstad et al. 2018). This is why the use of grazing organisms is so important, as they live in the benthos and feed on benthic biofilms. Several toxicity tests have been conducted in human cell culture systems (e.g. Ahamed et al. 2020; Siddiqui et al. 2013; Ahamed et al. 2013; Ahamed et al. 2019; Haase et al. 2012; Mrakovcic et al. 2013; Lategan et al. 2018). A few studies have focused on aquatic animals that could represent different habits or feeding behaviour that address and describe the fate characteristics of sedimentation and agglomeration (Abdel-Tawab et al. 2022; Jenifer et al. 2020; Malhotra et al. 2020). It should also be mentioned that there are very few studies on the impact of *weathered* nanomaterials on organisms (Politowski et al. 2021a, 2021b). However, weathering processes differ depending on the environmental compartments, and the resulting differences in the degradation processes of the nanotubes reflect these environmental conditions.

Uptake of ingested wMWCNTs into digestive or absorptive cells plays a major role but little is known about the fate of wMWCNTs in absorbing epithelial tissues of invertebrates for lentic and lotic water ecosystems. Therefore, we conducted a histological study for *L. stagnalis* and *R. semicolorata.* With this study, we aim to investigate the fate of ingested wMWCNTs in freshwater grazers, which are suitable organisms to provide a deeper insight based on histological and physiological studies to better target risks.

## Materials and methods

### Microcosm

All experiments took place in air-conditioned laboratories at a constant temperature of 20 ± 1 °C with a light/dark cycle of 12/12 h for both species. Oxygen was introduced at 16 L/min via a Pasteur pipette, which was mounted on a tube and an air pump (Hailea Aco 9630) to ensure not less than 60% of oxygen during the whole experiments. The experimental procedure with *L. stagnalis* was realized for 24 days of exposure with 10 mg/L wMWCNTs with a subsequent depuration of 28 days. This study was divided into physiological and histological methods and is described in detail by Weise et al. (2021).

The experiments with *R. semicolorata* were performed with an exposure time of 28 days, and different concentrations of wMWCNTs were used (0.1 mg/L, 1 mg/L and 10 mg/L), but without a depuration time. Each aquarium contained one stone, nine larvae of *R. semicolorata* and six biofilm-covered microscope slides. For the exposure aquaria, 1 L of the wMWCNT-solution was added, according to the concentration level. Five aquaria were used for control and each concentration level of the exposure (0.1 mg/L, 1 mg/L and 10 mg/L wMWCNTs). The benthic biofilm was sampled in the Gauernitzbach, a second-order mountain stream of 4.6 km length and tributary of the River Elbe. After 1 week of attachment and growth, slides containing benthic biofilm were randomly removed and transferred to the experimental aquaria. Aquaria were tiled with 30 microscope slides (Roth, Karlsruhe–Germany) consisting of lime-natron-glass (76 × 26 mm) with a thickness of 1 mm.

### Test substance wMWCNT

The applied test substance wMWCNT (Baytubes C 150 P, BTS, Leverkusen, Germany) was synthesized and purchased from Bayer MaterialScience AG 2007 and is described precisely by Politowski et al. (2021a, 2021b) and Weise et al. (2021).

### Test organism L. stagnalis and R. semicolorata

Living individuals of *L. stagnalis* were obtained from the breeding station INRA (French National Institute for Agricultural Research, France) for the experiments. They were reared in Borgmann medium according to the recipe of LO-4S E + H (Borgmann 1996) and fed 3 to 4 times a week with small pieces of organic cucumber and organic salad. *R. semicolorata* were collected by kick sampling (Whitehurst and Lindsey 1990) and stone picking in the lower mountain stream Gauernitzbach. After sampling, 300 organisms were kept in plastic boxes and cooling boxes with stones and water for transportation to the laboratory at the Institute of Hydrobiology. For acclimatization, the larvae were kept in the transport boxes with aeration (6 °C) for 1 day, and after that, the mayflies were adapted 3 weeks to Borgmann medium modified with one-eighth of the amount of CaCO_3_ (Borgmann 1996; Kroll et al. 2016; Rybicki et al. 2012) and fed with benthic biofilm to ensure the best possible adaptation. The medium in the aquaria was renewed once a week with fresh Borgmann medium (see Weise et al. 2021). Only healthy and mid-sized larvae organisms without black wing-pads were used for the experiments because shortly before emergence, larvae cease feeding (Winkelmann et al. 2014).

### Electron microscopy and histology

For transmission electron microscopy (TEM) and histology, specimens were prepared as recently described (Weise et al. 2021). For each experimental group, 3–5 animals were processed and examined. Briefly, snails were sedated in 1% hydroxylamine solution, then the shell was removed and the animals fixed in 4% formaldehyde in 100 mM phosphate buffer, including postfixation with modified Karnovsky fixative (Karnovsky 1965). The step of decalcification was realized with 20% aqueous EDTA, postfixation with Osmium-TCH-Osmium (OTO), en bloc contrasting with 1% aqueous uranyl acetate, dehydration in a graded series of ethanol and infiltration and embedding in the epon substitute EMBed 812. Semithin sections were stained with toluidine blue/borax, and ultrathin sections were contrasted with lead citrate and uranyl acetate. The larvae of *R. semicolorata* were treated likewise except that decalcification was omitted. Semithin sections were imaged with a Keyence Biozero 8000 light microscope, and ultrathin sections were analysed with a Jeol JEM 1400Plus TEM (JEOL, Freising, Germany, camera: Ruby, JEOL) running at 80 kV acceleration voltage.

### Sublethal effects

Phagosomes in enterocytes were quantified by counting in the cytoplasm of columnar cells dominating the midgut of *R. semicolorata*. Phagosomes were counted only in fully visible digestive cells in TEM images.

### Analysis of physiological markers

Glycogen, triglycerides (TGs) and the ratio of RNA/DNA were measured at the University of Koblenz-Landau. For *L. stagnalis*, 4 replicates from each sampling point (3 snails pooled) were used to analyse all mentioned physiological markers. After the removal of the shells, all animals were freeze-dried for 24 h (Shimadzu Emit, Christ GDH-60, Series: 603,876). Dry masses (3 pooled samples per replicate) of *L. stagnalis* and *R. semicolorata* were then examined to determine if there were significant differences in weight. Additionally, larval lengths were also examined (see data in Supplementary Information, SI).

Larvae of *R. semicolorata* were placed in 1.5-mL Eppendorf tubes and then transferred to liquid nitrogen. After that, pooled samples of three individuals and four replicates for each for the controls and the exposure treatments were freeze-dried for 24 h (Christ Alpha 1–2, Osterode am Harz, Germany). Afterwards, the samples of *R. semicolorata* were weighed with a microbalance (Secura 225D, Satorius, Göttingen–Germany) and homogenized in a bead mill (Retsch 40MM, Hahn–Germany) with glass beads for 2 × 3 min at 25 Hz. Glycogen and TG contents of pooled mayfly samples were determined by enzymatic assays, using a sequential approach due to low biomasses (< 3 mg). For glycogen, the samples were extracted in perchloric acid (PCA). After that, a neutralization with KHCO_3_ (2 M) was performed, and glycogen was hydrolysed using amyl glucosidase (Sigma-Aldrich, Steinheim, Germany); see Hoppeler et al. (2017). Thereafter, TGs were extracted and analysed with an enzymatic assay by using ice-cold hexane as extracting agent (Hoppeler et al. 2017). Photometric determination of TG concentration was performed using a commercial TG assay (Triglyceride FS, DiaSys Diagnostic Systems, Holzheim, Germany). In the enzymatic reaction, the lipoprotein lipase splits TG into glycerol and free fatty acids (for further description, refer to Weise et al. (2021)). The RNA/DNA ratio was measured for a total sample of more than 5 mg. Total nucleic acids were extracted using the MasterPure™ Complete DNA and RNA Purification Kit (Epicentre, Madison, USA) following the protocol for tissue samples (EPICENTRE 2012, Ch. 6 A and B). Cells were digested by adding proteinase K, tissue and cell lysis solution (1:300) and homogenized twice (bead mill, 5 min) before incubation at 65 °C for 15 min in a bio shaker. After stopping the enzymatic reaction on ice (3–5 min), precipitation of proteins was initiated with 150 µl of precipitant, brief vortexing (10 s) and centrifugation for 10 min at 18,600 × g and 4 °C. The supernatant was transferred to a sterile RNase-free Eppendorf tube (1.5 mL).

### General experimental set-up

**Fig. 1:**
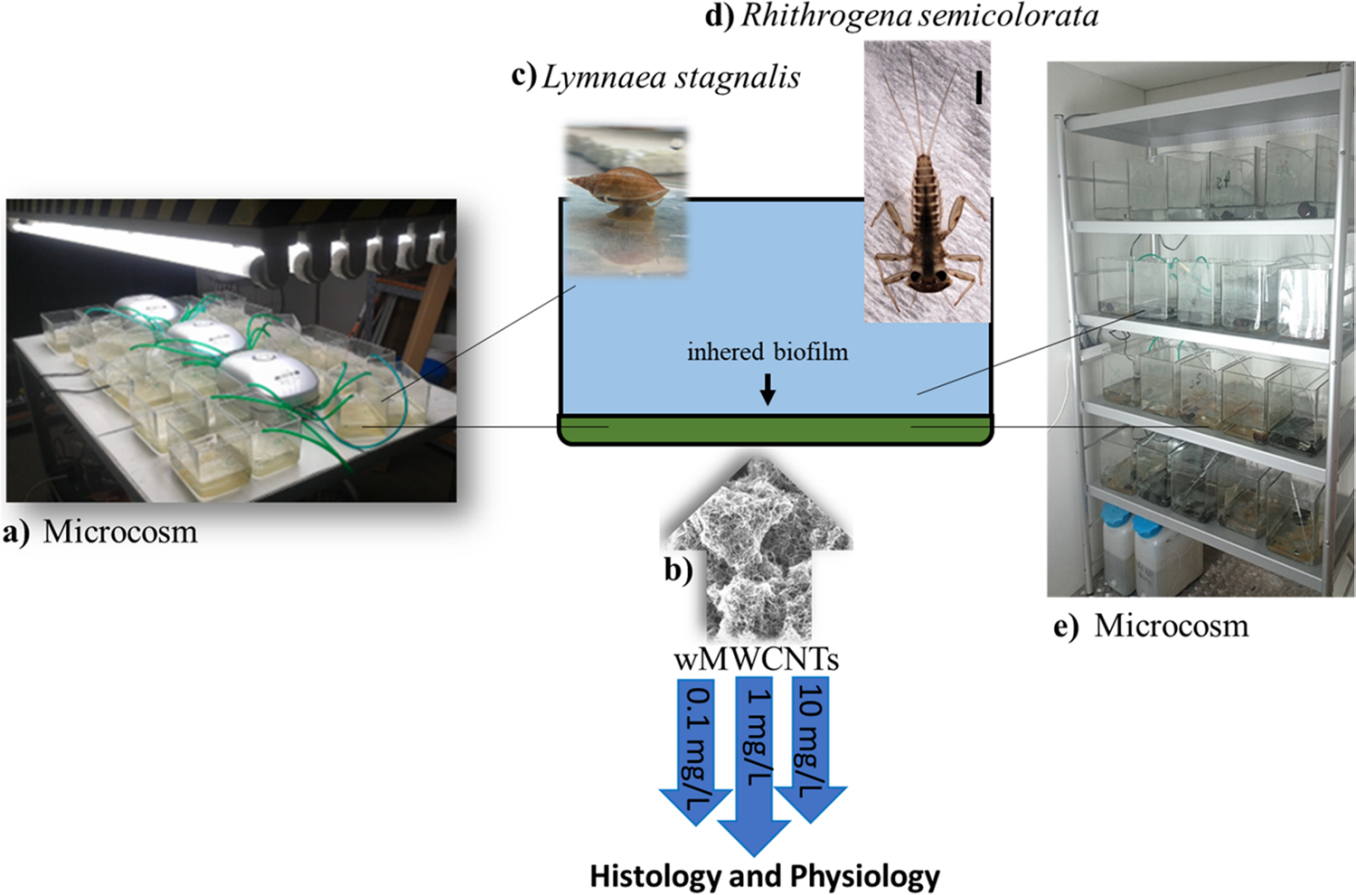
Overview of the experimental set-up: a) microcosm for *L. stagnalis*, b) wMWCNTs (SEM), c) *L.* *stagnalis*, d) *R. semicolorata* (alive image), e) microcosm for *R. semicolorata*. Chosen methods are indicated with arrows (blue)

For the test organisms used (Fig. 1), a mortality assay was performed in parallel. The study was performed with 10 mg/L wMWCNTs during 24 days with four replicates of *L. stagnalis* (see Weise et al. 2021) and 28 days for *R. semicolorata* with five replicates (each pooled with three samples). This study was divided into histology and physiology methods. Controls were examined parallel to all methods.

### Statistics

Data analysis was performed with the R software (RStudio Team 2017 and R Core Team 2018). Wilcoxon signed rank test was used for physiological markers, which is applicable for outlier analysis and thus resistant to aberrations. Concerning the results of phagosomes per cell, an ANOVA test design was chosen. For that, data of the endpoint “number of phagosomes” were Box-Cox transformed to correct inhomogeneous variances (Box and Cox 1964). No observed effect concentrations (NOECs) and/or lowest observed effect concentrations (LOEC) were calculated, using the outputs of contrast analyses. Weight and length measurements (Supplementary Information, SI) were calculated using a one-factor analysis of variance (ANOVA) to determine statistically significant differences between control and exposure treatments.

## Results and discussion

### Histology and electron microscopy of *L. stagnalis* digestive gland

**Fig. 2:**
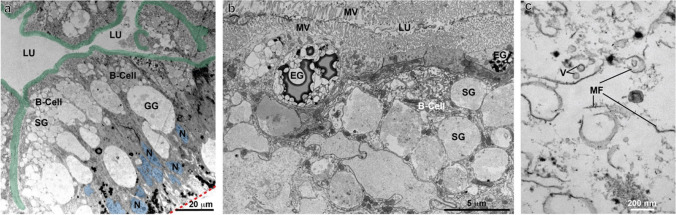
TEM-micrographs of digestive gland in control animals. **(a)** The digestive epithelium with B-cells which contain many phagocytic granules of different sizes (e.g. small granules, SG and large green granules, GG). The microvilli are pseudo-coloured in green; the nuclei are indicated in blue and the basement membrane as a red dashed line. **(b)** Apical parts of B-cells with phagocytic granules (SG, GG) and interspersed D-cells with excretory granules (EG), the tight lumen (LU) and the microvilli (MV) are shown. **(c)** A green granule at higher magnification, filled with cellular debris such as vesicles and membrane fragments (MFs)

The digestive gland of *L. stagnalis* is composed of diverticula with branched tubules lined with a single-layered digestive epithelium (Fig. 2). The food is transported repeatedly into these tubules for digestion and resorption. TEM analysis revealed mainly digestive B-cells and a few excretory D-cells and proliferative A-cells. The resorptive B-cells are characterized by microvilli at the apical surface, many apical phagocytotic granules (small granules, SG) that fuse into larger granules such as the green granules (GG) (Fig. 2). In addition, multiple mitochondria and multivesicular bodies can be seen, indicating ATP-driven transport processes and active endocytosis. For example, Moore (2006) described an intracellular uptake of MWCNTs as most likely to occur by endocytosis in midgut digestive cells. Close inspection of small and green granules revealed mainly cellular debris and membrane fractions in them (Fig. 2c). These are the sites where wMWCNTs are most likely to be found if they are resorbed upon ingestion of wMWCNT-contaminated biofilm.

**Fig. 3:**
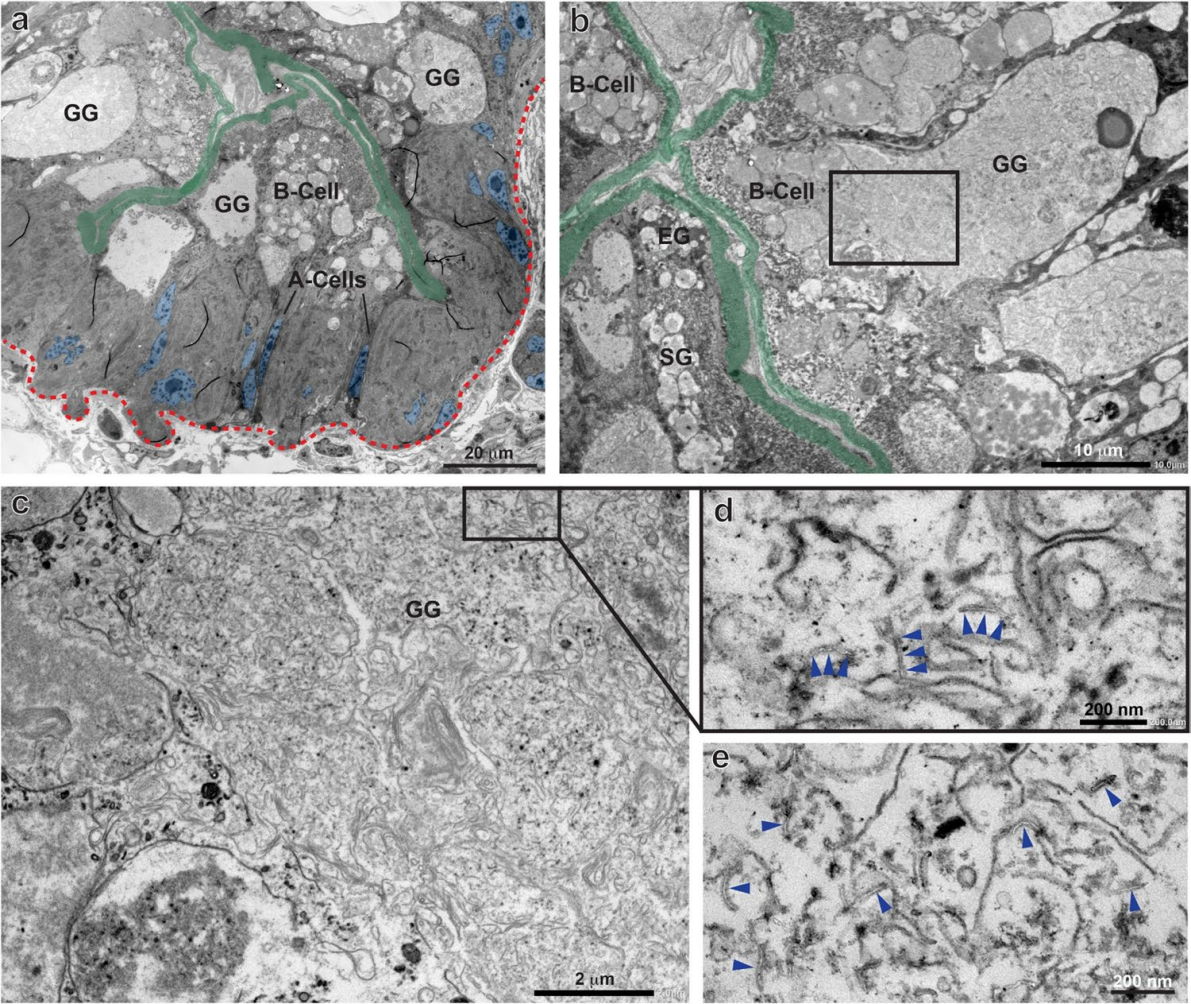
TEM micrographs of the digestive gland after feeding with wMWCNTs. **(a)** Overview of the digestive gland epithelium, apical microvilli and the basement membrane are indicated in green and by a dashed red line, respectively. B-cells with phagocytic granules, and 2 A-cells are indicated. **(b)** Digestive gland epithelium at higher magnification; a B-cell with a large GG is shown. The square indicates the region shown in C. **(c)** A large GG at higher magnification with a lot of cellular debris and membrane swirls, the square indicates the area shown in d. **(d)** GG content at higher magnification with wMWCNTs (arrowheads) among membrane fragments. **(e)** Another example of wMWCNTs (arrowheads) in a phagocytic granule

**Fig. 4:**
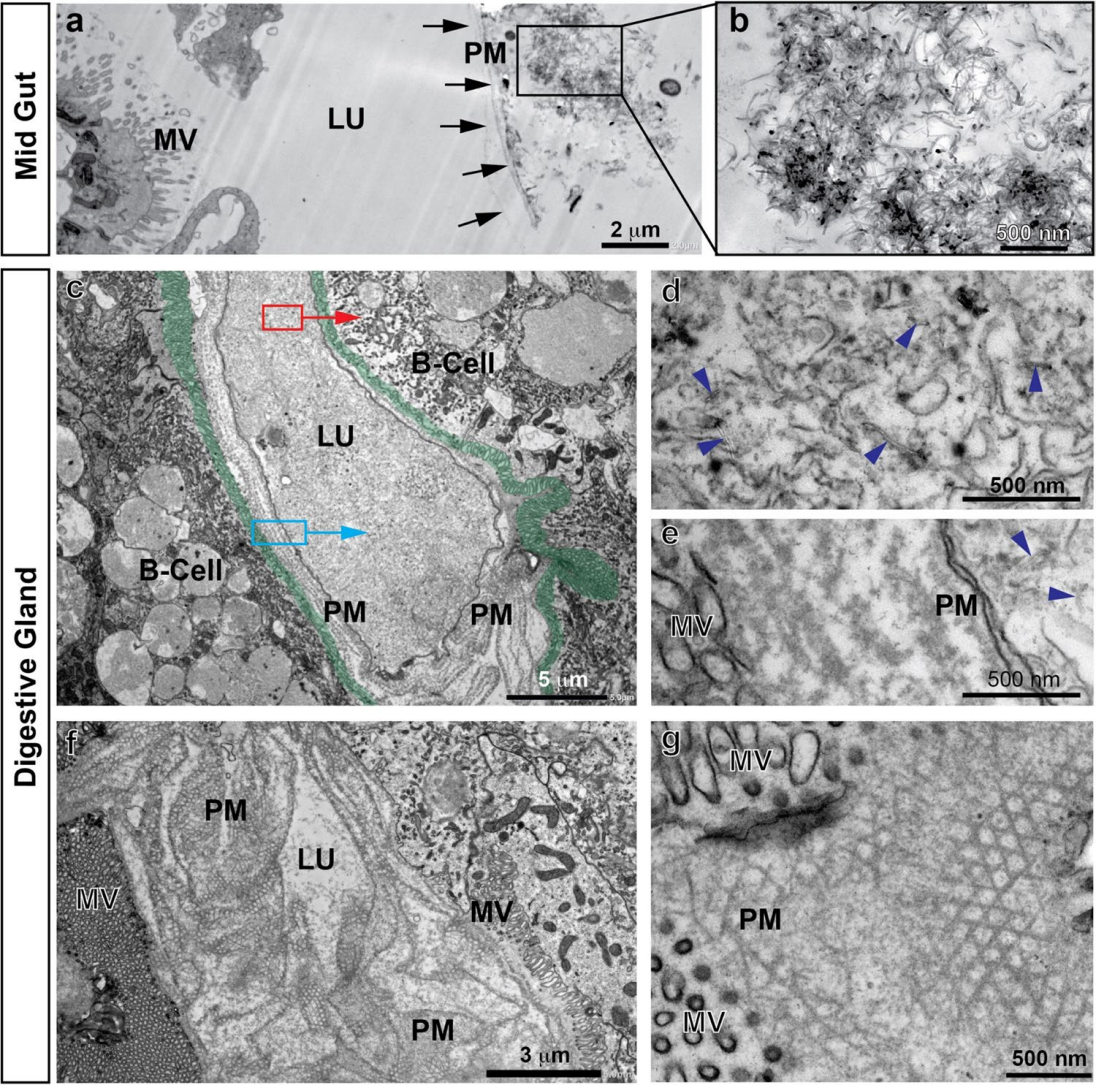
Peritrophic membranes (PMs) in the midgut (a, b) and in the digestive gland (c–g). **(a, b)** The PM (arrows) separates the apical surface of the gut epithelium from the food bolus which also contains bundles of wMWCNTs. **(c)** Digestive gland duct with epithelial cells (apical microvilli pseudo-coloured in green), and a lumen, filled with material, and separated from the epithelium by a multilayered PM. The red and blue squares indicate the regions shown in d and e, respectively. **(d, e)** wMWCNTs (arrowheads) in the lumen at higher magnifications

Snails fed on biofilm with wMWCNTs displayed a normal digestive gland organization (Fig. 3). The wMWCNTs were found either in the lumen of the digestive gland tubules or in sparse patterns in the phagocytic granules of B-cells, either in the GG or rarely in the SG (Figs. 3 and 4). In the midgut lumen, large bundles of wMWCNTs can be found (Fig. 4a, b, see also Weise et al. 2021), but no wMWCNTs could be detected in its epithelial cells. This is consistent with observations by Petersen et al. (2009) who showed the presence of MWCNTs in the intestinal lumen of *D. magna*, but no uptake into the cells. In the digestive gland of snails, only single wMWCNTs were seen in rare occasions (Fig. 4c, d, e), and a few could be detected in B-cell granules (Fig. 3c, d, e). Thus, there was cellular uptake, but only a limited fraction of wMWCNTs made their way through the alimentary canal into the digestive gland cells and into the digestive granules. Obviously, the majority of wMWCNTs were prevented from reaching the site of resorption in the digestive gland. The few individual wMWCNTs that make it into the lumen and cells do not cause serious damage, based on the normal morphology of the digestive gland cells. This is consistent with measurements of dry weight before and after feeding as well as after depuration, which all showed no significant differences (Supplementary Figure 1).

One potential mechanism to protect epithelial cells in the intestinal tube and the digestive gland from mechanical or chemical damage is a peritrophic membrane (PM) (Sadava et al. 2018). Although a well-described feature in insects and other arthropods, much less is known about PMs in molluscs (Peters 1969; Valk et al. 2014). The PM surrounds the food bolus in the intestine and is made by protein and/or sugar fibres to form a filter-like structure. In the lumen of *L. stagnalis* midgut, it forms a multilayered filter membrane that prevents wMWCNT bundles or other bulky biofilm material to contact the gut epithelium (Fig. 4a, b). In the digestive gland, the PM can be seen in close association with the microvilli, very often in several layers (Fig. 4c, e, f). In some sections, the lattice-like structure of the PM becomes apparent (Fig. 4g). The PM might be the structural basis for the protection of the intestinal epithelium against wMWCNTs and might help to explain why the mud snails tolerate a wide range of wMWCNT concentrations. Finally, after 28 days of depuration, no wMWCNTs were detected in the gut or digestive glands. Thus, we show the fate of wMWCNTs entering the cells of *L. stagnalis*, but after a period of depuration, the snails are able to recover (see also Weise et al. 2021). There might be greater risk if there is no depuration phase, and the wMWCNTs are ingested over a long period of time. This could lead to greater accumulation in the cells, possibly associated with metabolic damage and consequently transport of wMWCNTs to higher trophic levels due to food web transfer. Thus, long-term accumulation studies would be needed to address this issue.

#### Histology and electron microscopy of *R. semicolorata* intestine

**Fig. 5:**
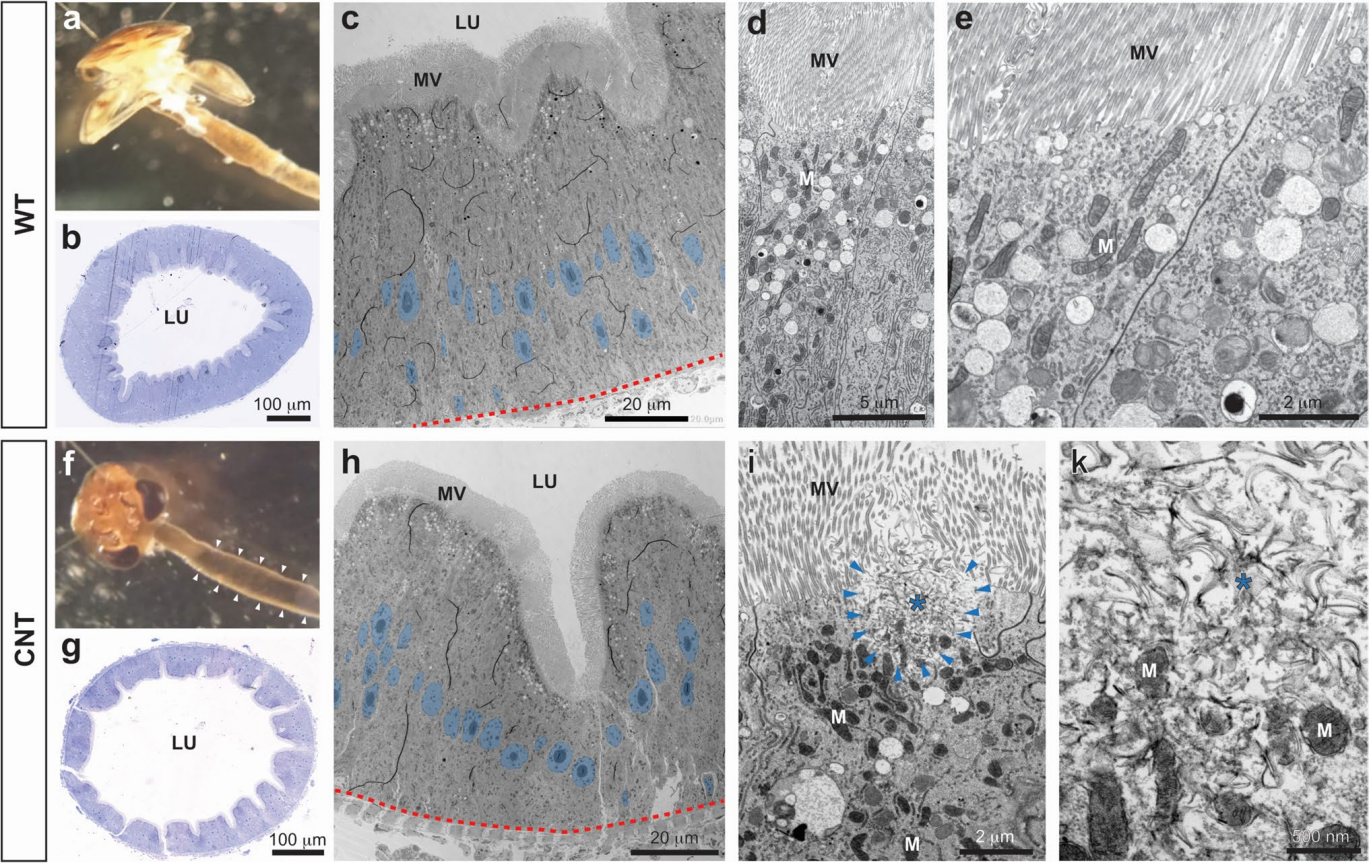
*R. semicolorata* intestine in control animals (WT, wild type) (a–e) and in animals exposed to 10 mg/L wMWCNTs (f–k). (a) the brown-coloured gut in a WT individuum; (b) semithin section through the midgut, LU, lumen; (c) EM overview image of the midgut epithelium; nuclei are indicated in blue, the basement membrane with a red dashed line, MV, microvilli; (d, e) apical part of the epithelial cells at higher magnifications, the MV, mitochondria (M) and phagosomes (PS) are indicated. (f) The midgut of an animal after 10 mg/L wMWCNTs, note the dark material in the gut after dietary uptake (arrowheads), (g) semithin section through the midgut; (h) EM overview image of the midgut epithelium; (i, k) apical surface of midgut epithelial cells at higher magnification; arrowheads indicate damages in the apical cytoplasm and microvilli, the asterisks indicate identical positions in i and k, respectively; mitochondria (M) and phagosomes (PS) are indicated

In the control group, the intestine is uniformly coloured in brown (Fig. 5a), and the midgut epithelium is dominated by the columnar digestive cells with prominent and homogeneous microvilli (Fig. 5b–e) and many apical phagosomes (Fig. 5d, e). In animals exposed to 10 mg/L wMWCNTs, the intestine contains a darkly coloured mass indicating the CNTs (Fig. 5f, white arrowheads). Overall, the midgut epithelium looks similar (Fig. 5g, h), but there are disturbances and damages in the microvilli and in the apical parts of the cells (Fig. 5h–k). These apical lesions are characterized by fragmentation of membrane structures both of the microvilli and of cytoplasmic organelles (Fig. 5i, k) and occur in just a few cells (arrows in Fig. 5h). Intracellular wMWCNTs could not be detected. Some of the membrane fragments resemble wMWCNTs but are of different diameter (compare Fig. 5k with Fig. 3d, e). These lesions were not observed in control animals. Taken together, wMWCNTs result in cellular damage, but the wMWCNTs themselves are not detectable at the damaged sites. The lesions may be therefore an indirect effect of wMWCNT contamination. In general, this could be due to nanomaterials being taken up and transported through the cell by endocytosis-related mechanisms (Van der Zande et al. 2020), which we, however, could not see. Theoretically, digestive enzymes, e.g., peptidases or lipases, could attack the polypeptide coatings or even be able to degrade organic components of the manufactured surface coatings of engineered nanomaterials (ENMs). Girardello et al. (2015) were able to observe internalization of MWCNTs in the muscle layer of leeches at concentrations of 400 mg/L. To validate the intracellular uptake of MWCNTs, they used a potassium hydroxide (KOH) digestion of the tissue. Therefore, the gross accumulation of MWCNTs in leech tissue was interpreted as entry through the skin. To further confirm this assumption, aggregated MWCNTs were detected in the muscle layer using TEM images. In our study, uptake of wMWCNTs could only be shown for the B-cells in the snail, but not in mayfly larva, potentially due to the PM working more effectively in insects than in gastropods. Aquatic insect larvae are described as often having a slow-moving peritrophic membrane surrounding the fast-moving gut contents (Wotton and Malmqvist 2001). Perhaps this is an explanation why wMWCNTs could not pass through the epithelial membrane. Although we cannot rule out some uptake of wMWCNTs in different areas of the gut, it is improbable that substantial amounts are reaching the tissue. This is also confirmed by measurements of dry weight and length of control and exposure animals (Supplementary Figure 2 and 3).

**Fig. 6:**
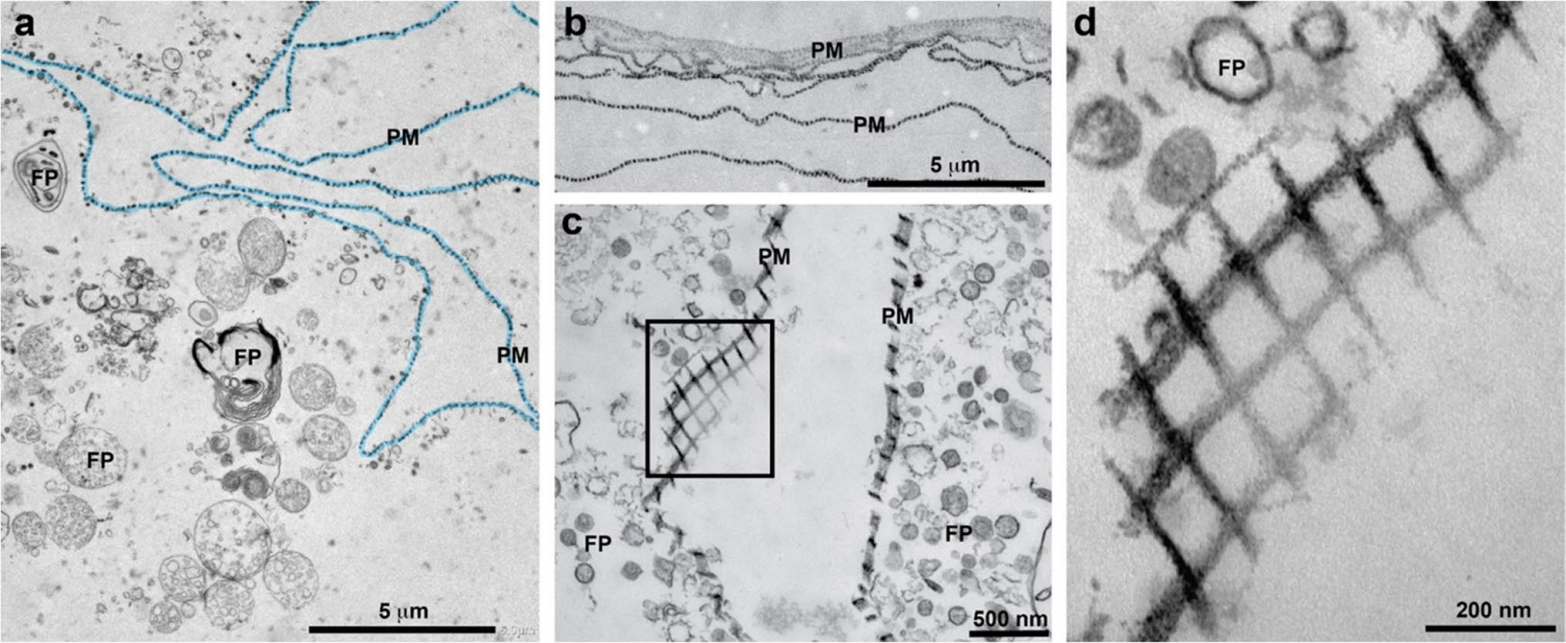
TEM investigations of PM in the midgut lumen of *R. semicolorata*. (a) Food particles and the PM; (b) Multiple layers of PM; (c, d) The PM at higher magnification cut at an angle, so that the lattice structure of the PM fence becomes apparent. The square indicates the region shown in d

As described above, the alimentary canal of insects is equipped with a PM that separates the midgut cavity with the food bolus (endoperitrophic space) from the surface of the epithelial cell in the midgut epithelium and the space above the microvillar brush border (ectoperitrophic space). One study investigated the role of PM in nanoparticle toxicity (Handy et al. 2012). Using TEM microscopy, they showed that gold nanoparticles attached to the microvilli of the intestinal cells of *D. magna*. Due to the preparation for EM which includes the dissection of the midgut tube, the PM appears often folded and multilayered, but the separation of food material on one side of the PM and the ectoperitrophic space beyond the PM is clearly visible in Fig. 6a and c. At higher magnifications, the lattice-like structure of the PM can be readily seen, formed square-shaped subdivisions with a rough length of 125 nm (Fig. 6c, d). The wMWCNTs in their own single strand have a length of about 1–10 µm and an outer diameter distribution between 5 and 20 nm. Therefore, bundles and even the majority of individual wMWCNTs could be retained by the PM. Comparing the structures of the respective PMs, it can be seen that it is more ordered and appears more erect in *R. semicolorata* than in *L. stagnalis* (Figs. 6 and 4). This could be the reason that in mayfly, the wMWCNTs could not penetrate the epithelial tissue that far in the first place, but only structurally altered the microvillus space.

#### Sublethal effects

**Fig. 7:**
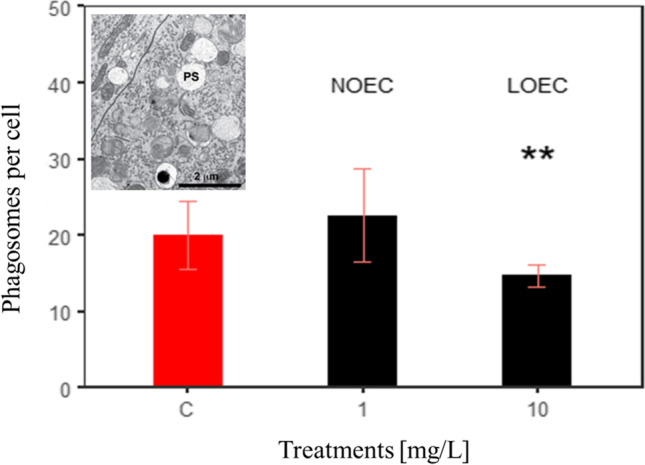
Results of phagosomes per cell (PS) with contrast analysis (ANOVA) after 28 days for *R. semicolorata* with (mean ± sd) for control, 1 mg/L and 10 mg/L wMWCNTs, p** < 0.01, *n* = 5

Changed levels of phagocytosis might indicate stress. Thus, we counted phagosomes in the cytoplasm of the columnar midgut epithelial cells, which are the dominant cell type in the midgut of *R. semicolorata* (Fig. 7). Contrast analyses showed significantly higher phagosome counts per cell for the control (20 ± 10.2) compared to 10 mg/L wMWCNTs (14.7 ± 10.2), which shows a value in the *F*-test (2, 101) = 6.85, *p* < 0.01). The highest number of phagosomes was counted in the treatment with 1 mg/L wMWCNTs (22.6 ± 9.2), which could be defined as the no observed effect concentration (NOEC). The highest concentration of 10 mg/L wMWCNTs caused the lowest number of phagosomes per cell and was determined as the lowest observed effect concentration (LOEC).

Phagosomes have the task of enzymatically degrading particles in the course of phagocytosis. Phagocytosis is an active transport process, which requires energy mostly in the form of adenosine triphosphate (ATP). Generally, when phagosomes fuse with lysosomes, macromolecules are enzymatically digested (endo- and exocytosis). The digestive cells contained high numbers of mitochondria and phagosomes with distinct electron-dense staining in the cytoplasm. This high number of mitochondria indicates high metabolic activity and transport activity of the cells. Thus, high numbers of phagosomes in the digestive cells indicate high lysosomal activity and storage capacity of nutrients (Dettner et al. 2010; Fent 2013). This is significant as exposed animals were unable to perform effective metabolic activity and lysosomal activity to store nutrients because they were stressed by the wMWCNTs. Thus, we show that at 10 mg/L wMWCNTs, *R. semicolorata* requires a lot of energy to provide ATP for phagocytosis, which might be obtained from stored energy (e.g., triglycerides).

#### Analysis of physiological markers

**Fig. 8:**
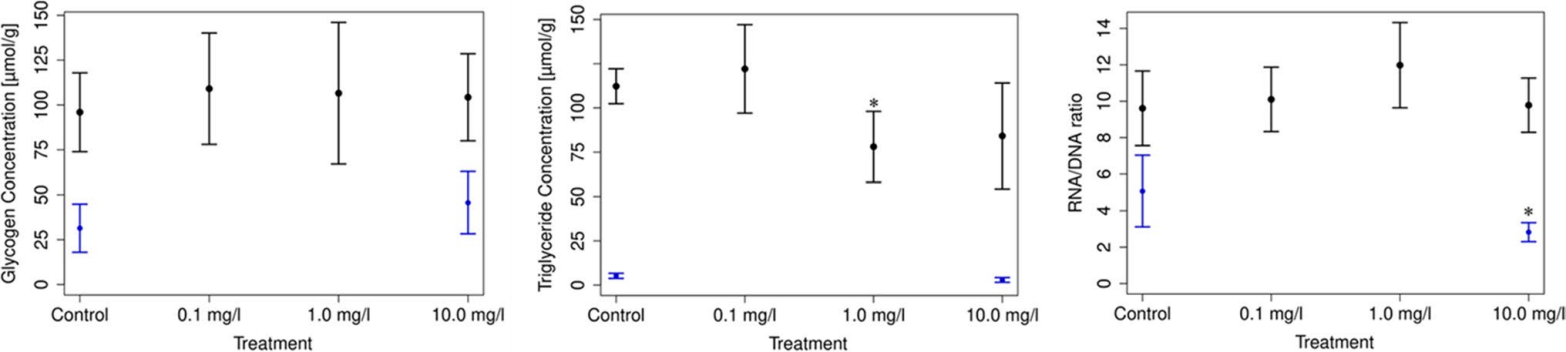
Left: Glycogen concentration (mean ± sd) after 28 days of the exposure in the controls and the treatments for *R. semicolorata* (black lined) and after 24 days for *L. stagnalis* (blue lined, from Weise et al. 2021). Middle: Results of the triglyceride analysis after 28 days (mean ± sd) of the exposure in the controls and the treatments for *R. semicolorata* (black lined) and after 24 days for *L. stagnalis* (blue lined). All concentrations of physiological markers (glycogen and triglycerides) are stated as “µmol per g (dry mass)” Right: Results of the RNA/DNA ratio after 28 days (mean ± sd) for *R. semicolorata* (black lined) and for *L. stagnalis* after 24 days (blue lined) are depicted, p* = 0.016 (black lined for *R. semicolorata*), p* for *L. stagnalis*)

Glycogen, TG concentration and RNA/DNA ratio of *R. semicolorata* after 28 days with 0.1 mg/L, 1 mg/L and 10 mg/L wMWCNTs (black lined) compared with *L. stagnalis* after 24 days of the exposure with 10 mg/L wMWCNTs (blue lined) (Fig. 8). Data for *L. stagnalis* were taken from Weise et al. (2021) for better comparison with *R. semicolorata*. For glycogen, no differences between control and treated animals are discernible for both species, but generally a higher glycogen concentration is present for *R. semicolorata* (Fig. 8a). Similarly, *R. semicolorata* has a very high TG content compared to *L. stagnalis* in both control and exposed animals. We can already show this in the control of *R. semicolorata* with a value of 112.3 ± 9.88 µmol/g, which then drops significantly to a value of 78.1 ± 19.99 µmol/g after 28 days of exposure to 1 mg/L (*p* = 0.016). The ingested wMWCNTs may have blocked and disrupted the digestive system of *R. semicolorata*, as has been observed, for example, in *Ceriodaphnia dubia* (Kennedy et al. 2008), which is in accord to the observed reduction in phagocytosis. At 10 mg/L, a mean TG concentration of 84.18 ± 29.92 µmol/g was observed. No significant differences can be found within the 5% significance level at 10 mg/L (*p* = 0.22), although the means show very similar values for 1 and 10 mg/L. This phenomenon, where the mean values of 1 mg/L are insignificantly different from 10 mg/L but still strongly different from the control (112.3 µmol/g), could possibly be attributed to the higher variance of the values at 10 mg/L (Fig. 8, middle). However, *L. stagnalis* showed low levels in TG concentration, which also showed a strong trend after 24 days of exposure to 10 mg/L wMWCNTs (Fig. 8, middle) (Weise et al. 2021). The situation is different with the RNA/DNA ratio. Here, no significant differences in the ratio can be detected at 0.1, 1 and 10 mg/L wMWCNTs after 28 days for *R. semicolorata*. However, *L. stagnalis*, revealed a significant difference in RNA/DNA ratio after 24 days of exposure to 10 mg/L of wMWCNTs. *L. stagnalis* revealed a very low TG content, whereas TG is significantly higher in *R. semicolorata.* This confirms that *R. semicolorata* are organisms with very high TG content (Winkelmann et al. 2007; Winkelmann and Koop 2007), indicating differences in metabolisms that are independent of wMWCNT treatment. The survival of all control organisms in our approach met the validity criteria in accordance with the OECD (2016) guideline no. 243, and no mortality in the control groups as well as for wMWCNTs exposed individuals of *L. stagnalis* occurred. Concerning *R. semicolorata*, the mortality did not exceed 10% during the whole experiment. In summary, the results and the conformity among the different controls means no impairments by the experimental test conditions concerning both organisms. Overall, the data show different effects in the respective organisms. The effect of wMWCNTs on *L. stagnalis* has already been explained in Weise et al. (2021) and shows here in this study that the wMWCNTs can penetrate into the cells but did not show a highly significant effect on TG concentration. This is possibly due to the already very low TG concentration of the animals. In addition to our physiological study, we also found no significant effects on the dry weight of *L. stagnalis* and *R. semicolorata* (see Supplementary Figures 1 and 2) and larval length measurements (see Supplementary Figure 3). In the case of *R. semicolorata*, however, there was a significant effect on the TG concentration, which was also reflected in the phagosome count. It can also be assumed here that in the case of depuration, the animals could no longer have any wMWCNTs in the tissue (was not investigated). Therefore, we conclude in general that PMs are the main reason for the low uptake into the tissues.

## Conclusion

We show here that wMWCNTs are able to enter the alimentary canal in two primary aquatic consumers: the snail *L. stagnalis* and the mayfly *R. semicolorata*, which both feed on benthic biofilms. After feeding, bundles of wMWCNTs were detected in the midgut lumina of *L. stagnalis* and *R. semicolorata*, whereas only single wMWCNTs were found in the digestive gland lumen of the snail. Intracellular uptake of wMWCNTs in *L. stagnalis* was restricted to the cells of the digestive gland, whereas no uptake into the absorbing cells was detected in the intestine of *R. semicolorata* larvae. Our histological results provide important insights into the behaviour of wMWCNTs in tissues of aquatic organisms. In addition, the RNA/DNA ratio was significantly altered in *L. stagnalis*, but not in *R. semicolorata*. In the mayfly, however, the triglyceride concentration was significantly reduced, and the number of phagosomes per enterocyte cell also decreased significantly. In both organisms, the peritrophic membrane may provide an important barrier to nanomaterials, and this may explain why only a few wMWCNTs were detected in the digestive gland cells of *L. stagnalis* and potentially in the microvilli fringe of *R. semicolorata*. Thus, the peritrophic membrane of these organisms might explain no lethal effects of wMWCNTs were observed for these species. This is the first histological study of wMWCNTs of grazing organisms in aquatic habitats and may trigger further research to investigate the potential risk of nanomaterials. In addition, the results of our study have important implications for the risk assessment of nanomaterials, which needs to be developed further, and the use of *L. stagnalis* and *R. semicolorata* should to be re-evaluated in light of the here presented results.

## Supplementary Information

Below is the link to the electronic supplementary material.

Supplementary file1 (DOCX 50.8 KB)

## Data Availability

On inquiry, the data presented in this study is available from the authors.
