# Supplementary material for: Impact of weathered multi-walled carbon nanotubes on the epithelial cells of the intestinal tract in the freshwater grazers Lymnaea stagnalis and Rhithrogena semicolorata
Source: Environ Sci Pollut Res Int. 2022 Jul 28;30(1):407–19. doi: 10.1007/s11356-022-22225-3 (PMC9813111; doi:10.1007/s11356-022-22225-3)
Supplement: Supplementary file 1 — Supplementary file1 (DOCX 50.8 KB) [file 11356_2022_22225_MOESM1_ESM.docx]

**Supplementary Information (SI)**

The following supplementary Figure 1 shows the weight trend of *L. stagnalis*. There are no significant differences between the respective groups. As a note, the t0 animals (reference) are the animals that were examined immediately after sampling. The control animals at day 24 compared to the exposure are almost in the same range, but for the depuration phase (animals exposed for 24 days followed by a 28-day depuration phase) an increasing trend can be seen compared to the exposure (day 24).


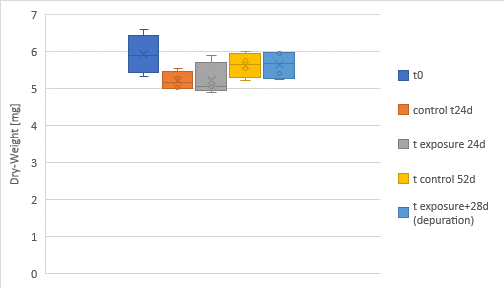


**Supplementary Fig.1** Results of the dry masses of *L. stagnalis* for t0, control (t24d and t52d) and exposure (t24d and texposure+28d) from Weise et al. (2021)

We have also investigated the dry weight and the lengths of *R. semicolorata* (supplementary Figure 2 and 3). Here we included 0.1 mg/L wMWCNTs in the analysis. The control animals as well as the exposed animals were each adapted after the t0 examination for 3 weeks at 6°C before the experiment was started. The comparisons of reference and control (as well as exposure) clarify that the animals adapted well to the system. There were also no significant differences in the length measurement (supplementary Figure 3).

**
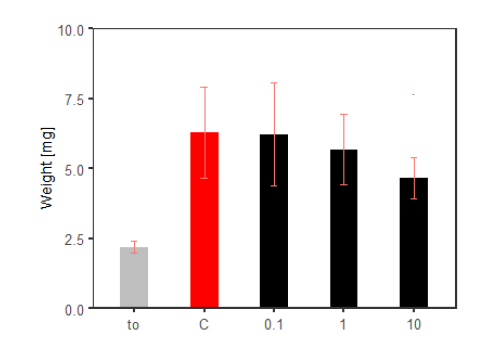
**

**Supplementary Fig.2** Results of the dry masses of *R. semicolorata* for t0, control exposure (0.1, 1 and 10 mg/L wMWCNTs)

**
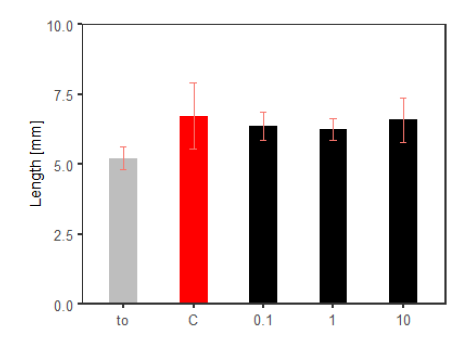
**

**Supplementary Fig.3** Results of the measured length of *R. semicolorata* for t0, control exposure (0.1, 1 and 10 mg/L wMWCNTs)
